# Group Dialectical Behavior Therapy for Binge Eating Disorder: Emotion Dysregulation and Alexithymia as Mediators of Symptom Improvement

**DOI:** 10.3390/nu17122003

**Published:** 2025-06-14

**Authors:** Luca Zompa, Emanuele Cassioli, Eleonora Rossi, Valentina Zofia Cordasco, Leda Caiati, Stefano Lucarelli, Ilenia Giunti, Lisa Lazzeretti, Giulio D’Anna, Simona Dei, Giuseppe Cardamone, Valdo Ricca, Francesco Rotella, Giovanni Castellini

**Affiliations:** 1 Psychiatry Unit, Department of Health Science, University of Florence, 50100 Florence, Italy; luca.zompa@unifi.it (L.Z.); emanuele.cassioli@unifi.it (E.C.); e.rossi@unifi.it (E.R.); valentinazofia.cordasco@unifi.it (V.Z.C.); leda.caiati@unifi.it (L.C.); valdo.ricca@unifi.it (V.R.); giovanni.castellini@unifi.it (G.C.); 2 Eating Disorders Unit, Central Tuscany Local Health Authority, 50142 Florence, Italy; stefano.lucarelli@uslcentro.toscana.it (S.L.); ilenia.giunti@uslcentro.toscana.it (I.G.); lisa.lazzeretti@uslcentro.toscana.it (L.L.); giulio.danna@uslcentro.toscana.it (G.D.); 3 Central Tuscany Local Health Authority, 50142 Florence, Italy; simona.dei@uslcentro.toscana.it; 4 Department of Mental Health, AUSL Toscana Centro, 59100 Prato, Italy; giuseppe.cardamone@uslcentro.toscana.it

**Keywords:** binge eating disorder, dialectical behaviour therapy, emotion dysregulation, alexithymia, psychotherapy

## Abstract

**Background/Objectives:** Dialectical Behaviour Therapy (DBT) has emerged as a promising intervention for Eating Disorders (Eds), especially Binge Eating Disorder (BED), which is often characterized by severe emotion dysregulation. The aims of this study were to evaluate the longitudinal course of BED symptomatology following a group-based DBT intervention focused on two specific modules, Emotion Regulation and Distress Tolerance, and to examine the mediating role of emotion dysregulation and alexithymia in symptom improvement. **Methods:** This non-randomized longitudinal clinical study involved 170 patients with BED who received a 16-week DBT group treatment including modules targeting emotion regulation and distress tolerance. Self-report questionnaires were administered at baseline (T0) and at the end of treatment (T1). Linear mixed models were used to analyze the longitudinal trend, and a mediation analysis was conducted to examine whether changes in emotion dysregulation and alexithymia mediated symptom improvement. **Results:** Longitudinal analyses showed a significant reduction in BED symptoms at the end of treatment as well as in the levels of emotion dysregulation and alexithymia. Mediation analyses revealed that both emotion dysregulation (indirect effect: −0.68 [−1.20; −0.31]) and alexithymia (indirect effect: −0.59 [−1.33; −0.20]) significantly mediated the improvement in BED symptoms over time. **Conclusions:** These findings support the application of focused DBT group interventions targeting emotion regulation and distress tolerance in reducing BED symptomatology. Emotion dysregulation and alexithymia were identified as mediators of longitudinal clinical improvement, highlighting the importance of modular and precision-based approaches in the treatment of BED.

## 1. Introduction

Binge Eating Disorder (BED) is the most prevalent Eating Disorder (ED) among the adult population [1,2]. According to the Diagnostic and Statistical Manual of Mental Disorders, 5th edition—Text Revision (DSM-5-TR) [3], BED involves recurrent episodes of binge eating defined as consuming an unusually large quantity of food within a short period of time while experiencing a sense of the loss of control, in the absence of regular compensatory behaviours. BED can be diagnosed if binge eating episodes occur at least once a week for 3 months [3]. Several studies underlined the importance of emotion dysregulation in the development, maintenance and persistence of EDs, including BED [4,5,6,7]. In particular, emotion dysregulation refers to patterns of emotion experience or expression that interfere with goal-directed activity [8,9,10]. It is not limited to the presence of intense or negative emotions but rather includes a broad range of difficulties in managing emotional states in a way that is flexible, adaptive and context-appropriate [11,12]. Emotion dysregulation may manifest as excessive emotion reactivity, difficulty recovering from emotion arousal, inability to modulate emotion responses or the use of maladaptive regulation strategies, such as suppression, avoidance or impulsive behaviour as seen in BED where individuals often engage in binge eating episodes as a dysfunctional attempt to manage overwhelming emotion states [13]. Closely related to the concept of emotion dysregulation is the construct of alexithymia, first introduced by Nemiah and Sifneos in the early 1970s [14,15] and characterized by difficulties identifying and describing feelings and an externally oriented thinking style. The defining features of alexithymia are in contrast to those of effective emotion regulation; furthermore, it seems that alexithymia appears to be a risk factor for several psychological disorders, including EDs, because it directly impairs emotion regulation [16,17,18]. Individuals with BED struggle significantly more with emotion awareness compared to both normal-weight and overweight individuals who do not have an ED diagnosis [19]. Moreover, individuals with BED perceive stressors more negatively and exhibit lower distress tolerance compared to healthy controls [13,20].

Given that recent reviews highlight only partially satisfactory response rates to commonly implemented treatments for BED, there is a recognized need to improve therapeutic outcomes [21,22]. Consequently, due to the strong association between emotion dysregulation and alexithymia with binge eating symptomatology [13,19], these constructs appear to be ideal candidates as mediating variables in the clinical improvement of patients with BED.

Dialectical Behaviour Therapy (DBT), originally developed for individuals with borderline personality disorder and chronic suicidal behaviour [23], targets pervasive emotion dysregulation and its behavioural consequences, thus representing a promising treatment for a wide range of psychopathological conditions sharing these psychopathological features [24]. Preliminary evidence supports its value in EDs characterized by dysregulated behaviours, i.e., Bulimia Nervosa and BED [25,26,27]. In particular, certain modules within DBT have been specifically developed to enhance emotion regulation, namely the Emotion Regulation and Distress Tolerance modules [28]. Although clinical guidelines recommend Enhanced Cognitive Behaviour Therapy (CBT-E) as standard treatment for BED [29,30], several studies have examined the application of DBT in the treatment of this disorder [31,32,33,34], highlighting how emotion regulation-focused intervention significantly reduced BED symptomatology particularly by decreasing the frequency of binge eating episodes [33,35] . In terms of efficacy, DBT for BED appears comparable to other therapeutic approaches, primarily CBT [32,36,37]. Regarding the use of DBT in group settings, a study conducted on a small sample of patients with BED demonstrated that symptom improvement remained stable at 3 and 6 months post-treatment [38]. Similarly, in a sample of 56 patients with BED who underwent 20 group DBT sessions in a community setting, a marked reduction in ED psychopathology was observed post-treatment, which was maintained at a one-month follow-up [39]. Another study found that even a brief intervention consisting of 10 group DBT sessions was as effective as a full-length DBT program in reducing binge eating episodes [40]. Despite the promising evidence supporting the use of group DBT for BED, significant heterogeneity exists among studies, and limited research has explored potential mediators underlying the therapeutic mechanisms and clinical improvement [22,31,41,42]. Given the central role of emotion dysregulation in BED, it is likely that the effectiveness of group DBT is largely attributable to its impact on improving emotion regulation abilities. However, the mediating role of improved emotion regulation in driving symptom reduction among patients with BED treated with group DBT has not yet been empirically demonstrated [42].

### Study Aims and Hypotheses

The present study aims to investigate the longitudinal course of BED symptomatology in patients undergoing group DBT, with a focus on the role of emotion dysregulation and alexithymia as potential mediators of symptom change over time. In particular, the Emotion Regulation and Distress Tolerance modules [28] were studied to characterize their impact, as these modules were specifically developed to target difficulties in emotion regulation and emotion recognition. We hypothesized that the DBT-based group intervention would lead to a significant reduction in binge eating symptoms, emotion dysregulation and alexithymia. Moreover, we expected that reductions in emotion dysregulation and alexithymia would independently mediate the longitudinal improvement in binge eating symptomatology.

## 2. Materials and Methods

This study was conducted within the Eating Disorders Treatment Network (EDTN) of the Florence area, which includes the Local Health Unit “Tuscany Centre” and the Eating Disorders Unit of the Florence University Hospital “Careggi”. Further details on the structure and functioning of the EDTN are available elsewhere [43]. Participants were recruited after providing informed consent for the use of their data for research purposes. The enrolment phase began in October 2016 and was completed in July 2021.

### 2.1. Ethical Considerations

All the diagnostic procedures and psychometric tests are part of the routine clinical assessment for patients with BED. Before the collection of data, the procedures of this study were fully explained; after that, the patients were asked to provide their written informed consent. This study was performed in line with the principles of the Declaration of Helsinki and was approved by the Institutional Ethics Committee of “Area Vasta Centro—CEAVC” (reference number: OSS.14.162).

### 2.2. Participants

The inclusion criteria were as follows: age > 18 years and a current diagnosis of BED according to the DSM-5 criteria in an acute phase of illness, as assessed by a clinical interview [44]. Exclusion criteria were as follows: intellectual disability, illiteracy, a current diagnosis of bipolar or psychotic disorder according to DSM-5 or absence of written informed consent.

### 2.3. Treatment

The treatment consisted of a group-based DBT program structured around two modules, Emotion Regulation and Distress Tolerance, as outlined in the DBT Skills Training Manual [28]. The treatment lasted 16 weeks, with one session of one hour per week, and included eight face-to-face sessions dedicated to each module. All sessions were held in dedicated public spaces within the healthcare facilities of the network. The Emotion Regulation module aimed to help patients better understand and manage their emotional experiences. Key components included psychoeducation on the function of emotions, the identification and labelling of emotional states, reducing emotional vulnerability through lifestyle strategies and the development of adaptive emotional responses such as opposite action and engagement in positive activities. The Distress Tolerance module focused on teaching individuals how to cope with acute emotional distress without resorting to impulsive or maladaptive behaviours, such as binge eating. The core skills taught included strategies to manage crisis episodes, such as TIPP skills (temperature, intense exercise, paced breathing, progressive muscle relaxation) which are designed to reduce emotional and physiological arousal. Distraction, self-soothing and radical acceptance are other essential tools that help patients tolerate distress and maintain behavioural control. In addition to the group DBT intervention, patients were included in a multidisciplinary treatment program that involved nutritional counselling and outpatient psychiatric consultations, in order to ensure comprehensive care. Each group was led by a clinical psychologist or psychiatrist and was co-facilitated by an assistant psychologist. All lead facilitators were DBT-trained and attended monthly peer DBT supervision sessions by trained psychotherapists who were experienced in EDs, in order to promote consistency, fidelity and treatment adherence. Participants were encouraged to apply the skills in their daily lives and complete homework assignments to reinforce learning. The treatment was specifically developed with the aim of enhancing emotion regulation and distress tolerance skills in order to reduce binge eating behaviours. The intervention was reported in compliance with the TIDieR (Template for Intervention Description and Replication) guidelines [45].

### 2.4. Study Design

The intervention was delivered as part of standard clinical practice in a naturalistic setting, in the context of a longitudinal non-randomized clinical study.

The clinical assessment was performed on the first day of DBT treatment, considered as the baseline time point (T0), and repeated at the end of treatment after the Distress Tolerance module (T1). Structured Clinical Interview for DSM-5 Disorders-Clinician Version, SCID-5-CV [44], was applied in order to identify the presence of comorbid psychiatric disorders, according to eligibility purposes. Self-reported questionnaires were administered to evaluate different psychopathological features, including the following:

- Binge Eating Scale (BES) [46]: The BES is a self-report questionnaires specifically designed to assess the presence and severity of binge eating symptomatology, and it was used in this study as the primary outcome measure. The scale consists of 16 items, each describing a set of statements that capture key behavioural and emotional aspects of binge eating episodes. These include the loss of control over eating, eating large amounts of food in a short time, feelings of guilt and shame, emotional distress related to eating and difficulties in stopping eating once started. Scores range from 0 to 46, with higher scores indicating a greater severity of binge eating pathology. The BES has demonstrated good psychometric properties, including internal consistency and construct validity [46], and it shows a moderate correlation with binge eating severity as assessed through food diaries and behavioural observations [47]. Cronbach’s Alpha was very good in the present sample (α = 0.89)
- Difficulties in Emotion Regulation Scale (DERS) [48]: A 36-item self-report questionnaire designed to assess multiple aspects of emotion regulation. The total score was analyzed, which is the sum of six subscale scores: Non-Acceptance of Emotional Responses, Difficulties Engaging in Goal-Directed Behaviour, Impulse Control Difficulties, Lack of Emotional Awareness, Limited Access to Emotion Regulation Strategies and Lack of Emotional Clarity. Higher scores indicate greater difficulty in emotion regulation. Internal consistency was good (α = 0.88).
- Toronto Alexithymia Scale (TAS-20) [49]: This scale is a widely used self-reported measure of alexithymia that assesses three factors: difficulty identifying feelings (Factor 1), difficulty describing feelings (Factor 2) and externally oriented thinking (Factor 3). Internal consistency was adequate (α = 0.75) in the present sample.

Participants completed the DERS, TAS and BES at the beginning and end of the treatment. The final assessment was conducted after 16 weeks, at the completion of the intervention, and it was administered as part of routine clinical practice, to monitor procedure effectiveness.

### 2.5. Statistical Analyses

Descriptive statistics were reported as the mean and standard deviation (SD). Adjusted linear mixed models with random intercepts were used to analyze the longitudinal trends in the outcome variables, with time and age included as fixed effects. The 95% confidence interval (CI) was computed for all time fixed effects. In addition, effect sizes (Cohen’s *d*) were calculated for all time effects to quantify the magnitude of the observed longitudinal changes. Patients who dropped out before the end of treatment were excluded from the analyses.

A two-instance repeated-measures mediation analysis was performed to verify whether time influenced BED symptoms through changes in emotion dysregulation and alexithymia. A single parallel longitudinal mediation model was used, with time as the within-subjects independent variable, emotion dysregulation (total DERS score) and alexithymia (total TAS score) as mediators and BED symptomatology (total BES score) as the dependent variable. Bootstrapping with 10,000 resamples was used to estimate the 95% confidence interval (CI) for indirect effects: an indirect effect was considered statistically significant if the CI excluded zero. Since the estimated relationship between the mediator and the dependent variable could not be assumed to be equal across instances of the independent variable in the two-instance repeated-measures mediation model, the interactions between time and the mediators were included in the models. This meant including the average variations in the mediators in the model, in addition to the differences. All statistical analyses were conducted using R statistical software version 4.5.0 [50]. Linear mixed models were created with nlme [51], whereas all mediation analyses were performed with lavaan [52], a dedicated package for path analyses within the R framework.

## 3. Results

### 3.1. The Characteristics of the Sample

The final sample consisted of 170 patients with BED, according to DSM-5 criteria (153 females and 17 males; mean age = 40.2, SD = 15.4), who completed all two evaluations and were therefore included in the final analysis. Participants were enrolled in a 16 week DBT-BED skill training group between October 2016 and July 2021. The mean BMI was 32.9 with SD = 12.0. The mean duration of illness was 16.1 years with SD = 10.9; 67% of the samples were workers, 15% were students and 18% were unemployed. Among all patients, 39.6% were diagnosed with comorbid mood disorders, including 60 with major depressive disorder and 4 with persistent depressive disorder. Furthermore, 13.6% had a comorbid anxiety disorder (*n* = 21), and 1.3% had a substance use disorder (*n* = 2).

### 3.2. Longitudinal Analysis

Table 1 shows the longitudinal trend in the psychometric variables. An overall improvement was observed across the investigated dimensions at the end of treatment, following the group DBT sessions (see Table 1). Statistically significant reductions were observed between baseline T0 and T1 for the DERS total score and those of its subscales: Non-Acceptance of Emotional Response, Difficulty Engaging in Goal-Directed Behaviour, Emotion Regulation Strategies, Lack of Emotional Clarity and Lack of Emotional Awareness (Table 1). However, no significant difference was found for the Impulse Control Difficulties subscale (Table 1). The TAS scale showed a significant reduction in its total score and in the Difficulty Identifying Feelings subscale (Table 1). No significant reductions were observed in the TAS subscale scores related to the descriptions of feelings and externally oriented thinking (Table 1). Finally, a significant reduction in binge eating symptomatology was observed, as reflected by the total BES score (Table 1).

### 3.3. Longitudinal Mediation Model

**Figure 1:**
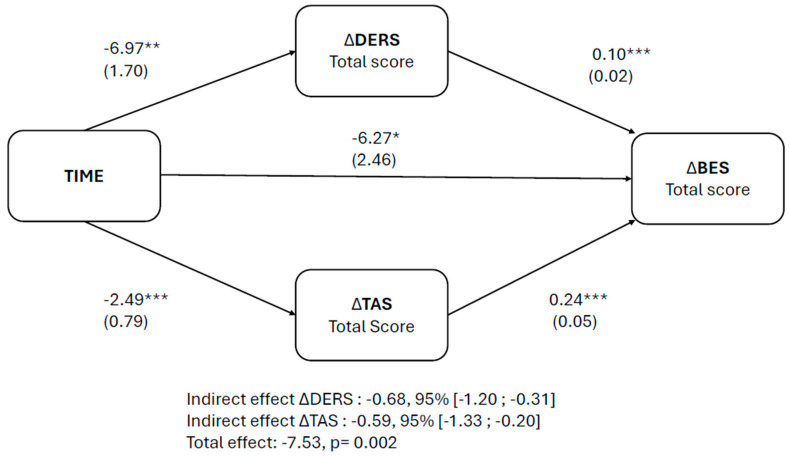
Longitudinal mediation model for BES. T1-T0 variations are indicated with Δ. TAS and DERS are considered as parallel mediators. Direct effects are represented as arrows with regression coefficients and standard errors in parentheses. DERS, Difficulties in Emotion Regulation Scale; TAS, Toronto Alexithymia Scale; BES, Binge Eating Scale. * *p* < 0.05; ** *p* < 0.01; *** *p* < 0.001.

A two-instance repeated-measures mediation analysis was performed to estimate the direct and indirect effects of the within-subjects independent variable, time, on the difference in BED symptomatology through the variation in both mediators, i.e., emotion dysregulation and alexithymia. The mediation analysis results are reported in Figure 1. Time had a significant total effect on the variation in the total BES score (Figure 1). The variation in the mediators significantly predicted the change in the outcome variable over time (Figure 1). Bootstrapping analysis showed significant indirect effects of time in terms of the total DERS score and total TAS score on BED psychopathology (Figure 1). However, time maintained its statistical significance even with the mediators as covariates, indicating a partial mediation with significant direct effects (Figure 1).

## 4. Discussion

### 4.1. Key Findings

The aim of the present study was to evaluate the longitudinal course of BED symptomatology through a group-based DBT intervention focused on two specific modules, Emotion Regulation and Distress Tolerance [28], in a large clinical sample of individuals diagnosed with BED. In addition, the mediating role of emotion dysregulation and alexithymia in symptom improvement was investigated. Specifically, the findings indicated that the therapeutic use of these two modules was associated with a significant reduction in BED symptomatology, achieved through improvements in emotion regulation and the ability to identify and recognize one’s emotional states. These results are consistent with previous evidence supporting the efficacy of DBT in the treatment of BED [34,38,53]. Our model showed a significant decrease in binge eating symptoms, both in terms of frequency and severity, with this reduction being mediated by enhanced emotion regulation strategies and emotional awareness. For the first time, this study examined emotion dysregulation and alexithymia as potential mediators of clinical outcome, emphasizing the importance of specifically targeting these two psychopathological dimensions. In patients with BED, difficulties in identifying and recognizing emotions may contribute to the onset of dysregulated behaviours, such as binge eating episodes, used as maladaptive coping strategies to manage negative emotional states [54,55,56,57]. Several studies have also highlighted that emotion dysregulation constitutes a transdiagnostic factor involved in the maintenance and relapse of EDs [58,59], suggesting that interventions targeting these difficulties may not only contribute to symptom reduction in BED [24,60] but also serve as protective factors against chronicity and support long-term recovery [4,61].

### 4.2. Clinical Implications

The clinical implications of these findings are particularly relevant. The implementation of brief DBT interventions focused on emotion regulation and distress tolerance represents an innovative aspect of this work, offering a cost-effective therapeutic option [62] that is easily applicable across various clinical settings. The brief and targeted form makes them particularly efficient and suitable within public health systems, where longer and more intensive formats may be harder to implement. Moreover, due to medical complications, individuals with EDs are at a higher risk of needing to access emergency services compared to the general population [63]. Gastrointestinal issues following binge eating episodes can frequently lead patients to seek urgent medical care. Interventions aimed at improving coping strategies for managing emotional crises may help reduce the need for emergency service utilization and promote a more appropriate use of healthcare resources. Future research may focus on this area to better understand its clinical and service-related implications. Given the relatively brief nature of the treatment tested in this study, it is reasonable to suggest that, within a framework of modular and targeted psychotherapy, such interventions could be integrated into broader and more structured approaches, such as CBT-E [64] in both individual and group formats. In line with transdiagnostic and dimensional models [65,66,67], the use of modular treatment approaches fits within the growing paradigm of precision psychotherapy, which emphasizes the construction of flexible personalized interventions tailored to core psychopathological processes while complementing traditional diagnostic frameworks [68,69,70]. Such integration becomes especially relevant for individuals with more treatment-resistant EDs, for whom interventions focused solely on eating-related cognitions and behaviours, as in standard CBT-E, may not be sufficiently effective [64,71,72]. In these cases, broadening the treatment scope to address key psychological dimensions such as emotion dysregulation and difficulties in emotional identification and processing may enhance therapeutic engagement, clinical response and relapse prevention. A notable example of an integrated approach was proposed by Rossi et al., who showed that trauma-focused interventions such as Eye Movement Desensitization and Reprocessing (EMDR), when used with patients with a history of neglect or maltreatment and combined with CBT-E, can significantly enhance treatment outcomes in eating disorders [57,73]. The inclusion of treatment modules targeting specific transdiagnostic mechanisms thus represents a key element in the personalization of care pathways, improving effectiveness according to individual patient needs [69,70,72]. The modular format lends itself to scalable training for clinicians, including those without extensive experience in DBT, thereby broadening access to evidence-based care. Additionally, these interventions may be particularly beneficial for patients who are ambivalent about longer-term therapy, offering a low-barrier entry point that can enhance engagement and retention. Future research should move in this direction by exploring the integration of diverse approaches based on specific psychopathological dimensions underlying BED.

### 4.3. Strengths and Limitations

A major strength of this study lies in the large sample size and the longitudinal mediation analysis conducted within a group DBT intervention for BED, a topic still under-investigated in the current literature [22,74]. Our findings display some limitations. The study design did not include a control group nor a direct comparison with other treatment approaches, thus limiting the generalizability of the results. Rigorous randomized controlled trials (RCTs) are needed in this field to confirm efficacy and establish proper cause–effect associations. Another limitation concerns the gender imbalance within the sample analyzed. Future studies should include long-term follow-up assessments to provide stronger evidence for sustained effects and directly compare brief group DBT interventions focused on selected modules with other established treatments for BED, for example, CBT-E and other therapeutic approaches. Larger samples and the use of advanced analytic methods, such as machine learning, may offer more precise insights into the mechanisms underlying the effectiveness of group DBT in this population.

## 5. Conclusions

In conclusion, this study supports the effectiveness of group-based DBT interventions focused on two core modules, Emotion Regulation and Distress Tolerance [28], in reducing BED-specific symptomatology. Clinical improvement was found to be mediated by enhanced emotion regulation capacities, including an improved identification and recognition of emotional states. In line with the framework of precision psychotherapy, the findings highlight the importance of designing personalized and targeted interventions for individuals with BED, using combined modular approaches capable of addressing the core psychological dimensions of this complex mental disorder.

## Figures and Tables

**Table 1:** Longitudinal trend in psychometric measurements.

Scales	T0	T1	Time Effect	Time Effect 95% CI	Cohen’s *d*
Total BES Score	26.36 ± 7.73	21.11 ± 7.78	−5.39 ***	−7.02–−3.76	−0.69
DERS: Non-Acceptance of Emotional Response	18.04 ± 6.68	15.26 ± 6.20	−2.40 **	−3.86–−0.95	−0.25
DERS: Difficulty Engaging in Goal-Directed Behaviour	18.00 ± 5.15	15.47 ± 4.95	−1.70 **	−2.98–−0.43	−0.18
DERS: Emotion Regulation Strategies	26.15 ± 5.02	22.50 ± 5.34	−3.18 ***	−4.55–−1.81	−0.45
DERS: Impulse Control Difficulties	19.09 ± 6.33	15.98 ± 5.72	−1.33	−2.83–0.17	−0.16
DERS: Lack of Emotional Clarity	16.29 ± 4.33	14.21 ± 4.23	−1.36 **	−2.36–−0.36	−0.26
DERS: Lack of Emotional Awareness	9.48 ± 3.35	8.16 ± 3.31	−1.23 **	−2.10–−0.36	−0.41
Total DERS Score	116.40 ± 25.42	100.26 ± 24.70	−11.15 ***	−16.64–−5.67	−0.41
TAS: Difficulty Identifying Feelings	23.10 ± 6.37	21.08 ± 6.23	−2.04 **	−3.52–−0.56	−0.27
TAS: Difficulty Describing Feelings	15.75 ± 4.47	15.32 ± 4.47	−0.44	−1.72–0.84	−0.13
TAS: Externally Oriented Thinking	19.59 ± 5.32	18.92 ± 5.33	−0.87	−2.10–0.36	−0.22
Total TAS Score	58.44 ± 12.17	55.32 ± 12.85	−3.42 *	−6.43–−0.40	−0.30

Abbreviations: DERS, Difficulties in Emotion Regulation Scale; TAS, Toronto Alexithymia Scale; BES, Binge Eating Scale. For each score, a statistical analysis was carried out using a linear mixed model, inserting time as a fixed effect with random intercepts. CI, confidence interval. This table reports the means and standard deviations for each time point, as well as the test results relating to the fixed effects (F). * *p* < 0.05; ** *p* < 0.01; *** *p* < 0.001.

## Data Availability

The data presented in this study are available on request from the corresponding author. The data are not publicly available due to privacy.
